# Apoptotic Gastritis in Melanoma Patients Treated With PD-1-Based Immune Checkpoint Inhibition – Clinical and Histopathological Findings Including the Diagnostic Value of Anti-Caspase-3 Immunohistochemistry

**DOI:** 10.3389/fonc.2021.725549

**Published:** 2021-08-11

**Authors:** Jan-Malte Placke, Josefine Rawitzer, Henning Reis, Jassin Rashidi-Alavijeh, Elisabeth Livingstone, Selma Ugurel, Eva Hadaschik, Klaus Griewank, Kurt Werner Schmid, Dirk Schadendorf, Alexander Roesch, Lisa Zimmer

**Affiliations:** ^1^Department of Dermatology, University Hospital Essen, University of Duisburg-Essen, Essen, Germany; ^2^Institute of Pathology, University Hospital Essen, University of Duisburg-Essen, Essen, Germany; ^3^Department of Gastroenterology and Hepatology, University Hospital Essen, University of Duisburg-Essen, Essen, Germany; ^4^German Consortium of Translational Cancer Research (DKTK), German Cancer Research Center (DKFZ), Heidelberg, Germany

**Keywords:** melanoma, immune check inhibitor (ICI), PD-1, gastritis, immune related adverse effects

## Abstract

**Background:**

Gastritis induced by checkpoint inhibitors (CPI) is a rare but severe drug-related side effect. The reference standard for confirming CPI-associated gastritis (CPI-assGastritis) is histopathological assessment; however, the histopathological features of CPI-assGastritis are not yet adequately defined.

**Materials and Methods:**

Gastric biopsies of melanoma patients with histopathologically suspected CPI-assGastritis were compared with gastric biopsies of patients with inflammation free gastric mucosa (IFGM), type A, B, and C gastritis with respect to apoptosis count and predominant histopathological features. Immunohistochemical anti-caspase-3 staining was performed to identify apoptosis. Quantification was performed by manually counting the number of apoptotic events per 10 high-power fields (HPF). Clinical symptoms, treatment, and follow-up data of patients with CPI-assGastritis were examined. The nonparametric Mann–Whitney *U* test was used for statistical testing.

**Results:**

Five melanoma patients (three women, two men; median age: 45 years) were treated with PD-1-based CPI. The patients reported epigastric pain, weight loss, nausea, and vomiting. Histologically, the patients with CPI-assGastritis showed a partly lymphocytic, partly granulocytic inflammatory infiltrate. Manual counting of apoptotic cells in biopsy tissue slides stained against caspase 3 revealed a median of 6 apoptotic events/10 HPF (95% CI, 2.75-17.30) in the patients with CPI-assGastritis. Results for the comparison cohort (patients n = 21) were a median of 1 apoptotic event/10 HPF (95% CI, 0.5–4.5) for type-A gastritis (six patients), a median of 2 apoptotic events/10 HPF (95% CI, 0–4.5) for type-B gastritis (five patients), and no apoptosis for IFGM and type-C gastritis (five patients). Patients with CPI-assGastritis had a significantly higher apoptosis count than patients with IFGM (p<0.01), type A (p<0.05), B (p<0.05), and C gastritis (p<0.01). None of the CPI-assGastritis biopsies showed evidence of *Helicobacter pylori*. All CPI-assGastritis patients responded to systemic treatment with corticosteroids.

**Conclusion:**

CPI-assGastritis manifests with nonspecific symptoms but histologically shows a high number of apoptotic events, which can best be visualized by anti-caspase-3 immunohistochemistry. This histopathological feature may help to histologically differentiate CPI-assGastritis from other forms of gastritis and inform decision-making regarding its optimal management.

## Introduction

The introduction of checkpoint inhibitors (CPI) as an anti-neoplastic treatment has led to an improvement in overall and progression-free survival for a wide range of tumors, e.g. non-small-cell lung cancer, Merkel cell carcinoma, and melanoma (1–3). Examples of CPI include programmed cell death protein 1 (PD-1), programmed cell death 1 ligand 1 (PD-L1), and cytotoxic T-lymphocyte-associated protein 4 (CTLA-4) antibodies. Furthermore, a combination therapy of nivolumab with the anti-CTLA-4 inhibitor ipilimumab is approved for treatment of non-small-cell lung cancer, renal cell carcinoma, and melanoma (4–6). Despite the improvement in progression-free and overall survival, up to 21% of patients treated with anti-PD-1-based monotherapy and up to 59% of patients treated with combined ipilimumab and nivolumab develop severe and life-threatening immune-related adverse events (classified according to the Common Terminology Criteria for Adverse Events [CTCAE]; grade 3/4) (7, 8). Common immune-related adverse events (ir-AEs) include colitis, hepatitis, thyroiditis, and cutaneous side effects. Rarer ir-AEs include myocarditis, nephritis, neurological AEs, and gastritis (9–11). Correct and early diagnosis followed by rapid treatment is crucial to prevent life-threatening AEs and permanent damage to patients (12).

Early and correct diagnosis of rare side effects such as autoimmune gastritis after CPI is especially difficult for several reasons. First, CPI-associated gastritis (CPI-assGastritis) is associated with nonspecific symptoms such as exhaustion, nausea, epigastric pain, vomiting, and weight loss. It also requires differential diagnosis from gastritis types A, B, and C, which have a high incidence in the general population. However, correct and early diagnosis is particularly important in the case of gastritis, because while steroids are the standard treatment for CPI-assGastritis, they are contraindicated for the common types of gastritis A, B, and C (13). Regarding patients who develop ir-colitis after CPI, it has been known for a while that inflammation is associated with prominent apoptotic features in glandular epithelia (14). In late 2019, Johncilla et al. described an increased number of apoptotic events as part of the histological pattern of CPI-assGastritis (15). Apoptosis is the process of regulated cell death. It is activated by an intrinsic or extrinsic signaling pathway. The intrinsic pathway is activated by intracellular stress in the mitochondria. In contrast, the extrinsic pathway is activated by external stimuli such as ligands of death receptors (16).

In daily clinical practice, no histopathological classification of CPI-assGastritis exists that clearly distinguishes it from common forms of gastritis such as types A, B, and C. The following study investigates whether apoptosis count is a good marker to differentiate CPI-assGastritis from frequently observed type A, B, and C gastritis. In addition, we evaluate patient and tumor characteristics, clinical symptoms, and treatment of patients with CPI-assGastritis.

## Patients and Methods

### Patient Data

Melanoma patients with histopathologically suspected CPI-assGastritis and patients with histopathologically confirmed inflammation free gastric mucosa (IFGM), type A, B, or C gastritis were included in the study. Histopathological diagnosis and tissue samples were provided by the Institute of Pathology of Essen University Hospital. Patient characteristics were obtained from the clinical database of Essen University Hospital. These characteristics included tumor stage, type of CPI, clinical symptoms, results of gastroscopy, therapy of side effects, and follow-up data from patients with CPI-assGastritis. All patients provided written, informed consent for their tissue to be examined. The study was approved by the ethics committee of Duisburg-Essen University and conducted in accordance with the Declaration of Helsinki. Human biological samples and related data were provided by the Westdeutsche Biobank Essen (WBE, Essen University Hospital, University of Duisburg-Essen, Essen, Germany, approval number 19-8705-BO).

### Immunohistochemical Staining and Examination

Archived, formalin-fixed, paraffin-embedded (FFPE) biopsies of patients with suspected CPI-assGastritis were taken from the biobank of Essen University Hospital. The biopsies were taken during the acute phase of the patient’s gastritis. Historical gastric biopsies of patients with confirmed IFGM, type A, B, and C gastritis, whose histopathological evaluation was complete, were used as a comparison cohort. These historical biopsies were taken from the Institute of Pathology, Essen University Hospital. The FFPE specimens were cut into 4-μm-thick consecutive slides of tissue. Routine hematoxylin&eosin (H&E) staining and anti-caspase-3 immunohistochemical staining were performed (Caspase 3, Cell Signaling Technology Inc.; Danvers, MA). Histochemical Giemsa staining was used to assess the presence of *Helicobacter pylori*. Immunohistochemical staining was performed using the Autostainer Link 48 (Agilent Dako; Santa Clara, CA).

The number of intraepithelial apoptotic events in the gastric mucosa in an area measuring 6.25 mm^2^ (10 HPF) was counted manually and in a blinded fashion by an advanced pathologist of the Institute of Pathology, Essen University Hospital. The apoptotic cells located in the glandular lumen were not counted, as they are physiologically present there in inflammatory altered gastric mucosa.

### Statistical Analysis

Statistical analysis was performed with GraphPad Prism (Version 8.4.3, GraphPad Software; San Diego, CA). For statistical testing, the nonparametric Mann–Whitney *U* test was performed. Two-sided p-values were evaluated, and a p-value of <0.05 was considered statistically significant.

## Results

### Patient Characteristics

A total of five melanoma patients (three women, two men) with histologically suspected CPI-assGastritis were included in the study. The comparison cohort comprised 21 patients: five with histologically confirmed IFGM, six with type-A gastritis and five patients each with type-B and -C gastritis. In the group with CPI-assGastritis, the median age of the patients at onset of symptoms was 45 years (range 38–54). In the group of patients with IFGM, gastric biopsies were taken at a median age of 57 (range 46-64), in patients with type-A gastritis, gastric biopsies were taken at a median age of 58.5 years (range, 49-75); in patients with type-B gastritis, gastric biopsies were taken at a median age of 43 years (range, 39-72); and in patients with type-C gastritis, gastric biopsies were taken at a median age of 52 years (range, 46-69). Patients with IFGM were female in 80% and male in 20%, type A gastritis were all female, and those with type B and type C gastritis were each 80% male and 20% female. All five CPI-assGastritis patients received combined CPI treatment (ipilimumab + nivolumab [n=3], pembrolizumab + epacadostat [n=1], nivolumab + relatlimab [n=1]) for stage IV metastatic melanoma. In addition, four of these five patients had other CPI-related ir-AEs, such as hepatitis, lichen ruber, or thyroiditis, and these occurred either shortly before or concurrently with gastritis (**Table 1**). All CPI-assGastritis patients manifested clinical symptoms, the most common of which were nausea and vomiting, epigastric pain, and weight loss. The median time between start of treatment and onset of clinical symptoms of CPI-assGastritis was 118 days (range 29–336 days). Detailed characteristics of the patients with CPI-assGastritis are listed in **Table 1**.

**Table 1:** Characteristics of melanoma patients with CPI-associated gastritis.

Patient	1	2	3	4	5
**Stage (AJCC, eighth edition)**	IV	IV	IV	IV	IV
**Age**	41	38	45	54	52
**Sex**	Female	Male	Male	Female	Female
**Other autoimmune events**	Hepatitis (concurrent with gastritis)	Lichen ruber (4 months before gastritis)	Hypophysitis Thyroiditis (2 months before gastritis)	Thyroiditis (3 months before gastritis)	None
**Weight loss**	None	9 kg in 3 w	10 kg in 3 w	8 kg in 3 w	None
**Clinical symptoms**	Nausea	Nausea	Heartburn	Nausea	
	Vomiting	Vomiting	Appetite loss	Vomiting	Hemoglobin loss
		Epigastric pain	Nausea		
**Time between start of CPI and occurrence of symptoms (days)**	29	336	118	166	50
**Last therapy before onset of gastritis**	Ipilimumab and nivolumab	Pembrolizumab and epacadostat	Nivolumab	Nivolumab and relatlimab	Ipilimumab and nivolumab
**Previous therapies**	None	None	Ipilimumab/nivolumab	None	BRAF/MEK inhibition

AJCC, American Joint Committee on Cancer; CPI, checkpoint inhibition; w, weeks.

### Endoscopic Results

**Figure 1:**
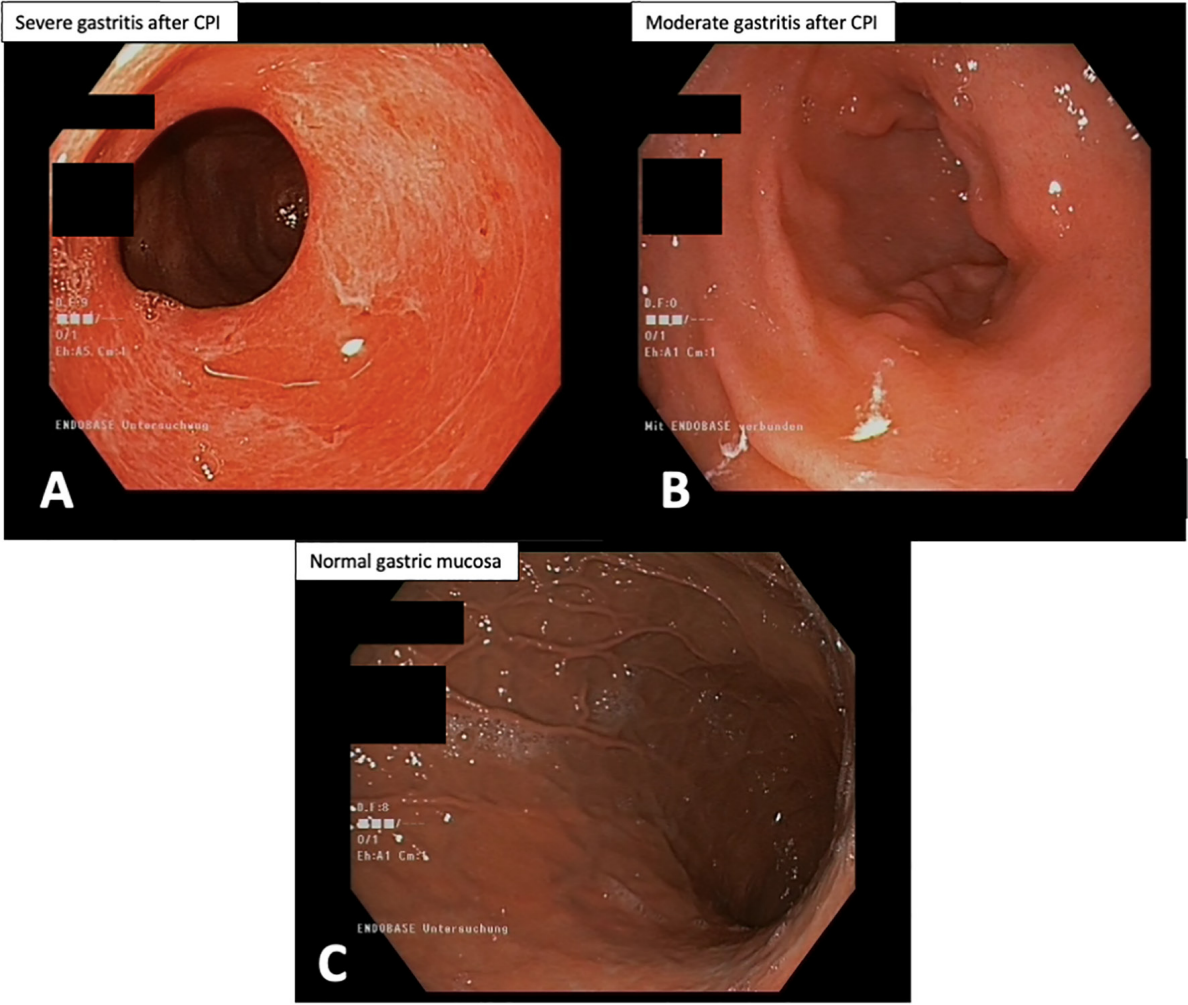
Examples of gastroscopy of two patients with CPI-associated gastritis. **(A)** Gastroscopy revealed erosive pangastritis and marked replacement of gastric mucosa. **(B)** Gastroscopy shows a case of a non-erosive pangastritis. **(C)** Gastroscopy shows normal gastric mucosa without inflammation. CPI, checkpoint inhibition.

A gastroscopy with guideline biopsies was performed on all five patients with CPI-assGastritis. The median time between onset of clinical symptoms and gastroscopy was 13 days (range 2–5 days). Macroscopically, four of the five patients CPI-assGastritis showed severe findings with partly edematous erythematous and partly hemorrhagic components in gastroscopy (**Table 2** and **Figure 1A**). One patient’s macroscopic finding was classified as moderate with partly striated erythematous mucosal changes (**Table 2** and **Figure 1B**). As a healthy comparison sample, **Figure 1C** shows the microscopic image of a patient with normal gastric mucosa.

**Table 2:** Clinical, endoscopic, and histological characteristics and clinical course of patients with CPI-associated gastritis.

Patient	Endoscopic features	Histopathological features	Treatment for ir-gastritis	Subsequent therapies	Autoimmune side effects after re-challenge
1	Moderate pangastritis	Lymphocytes Plasma cells Granulocytes Erosive mucosa 17 apoptoses/10 HPF	Pantoprazole 40 mg 2×/day Prednisolone 1 mg/kg bw	Dabrafenib plus trametinib	No re-challenge with CPI
2	Severe erosive pangastritis	Lymphocytes Plasma cells Granulocytes 4 apoptoses/10 HPF	Pantoprazole 40 mg 2×/day Prednisolone 1 mg/kg bw	Nivolumab plus relatlimab	No immune-related adverse events
3	Severe erythematous pangastritis	Lymphocytes Plasma cells Granulocytes Ulcerations 6 apoptoses/10 HPF	Pantoprazole 40 mg 2×/day Prednisolone 1 mg/kg bw	Pembrolizumab plus domatinostat	No immune-related adverse events
4	Severe pangastritis, separation of the gastric mucosa	Lymphocytes Plasma cells Granulocytes 14 apoptoses/10 HPF	Dexamethasone 8 mg Metoclopramide 10 mg 2×/day Pantoprazole 40 mg 2×/day	Nivolumab	No immune-related adverse events
5	Severe hemorrhagic pangastritis	Lymphocytes Plasma cells Granulocytes Intestinal metaplasia 3 apoptoses/10 HPF	Pantoprazole 40 mg 2×/day Prednisolone 1 mg/kg bw	Nivolumab Binimetinib plus encorafenib	No immune-related adverse events

CPI, checkpoint inhibition; ir, immune-related. bw, bodyweight; HPF, high power field.

### Conventional Histochemical Staining

**Figure 2:**
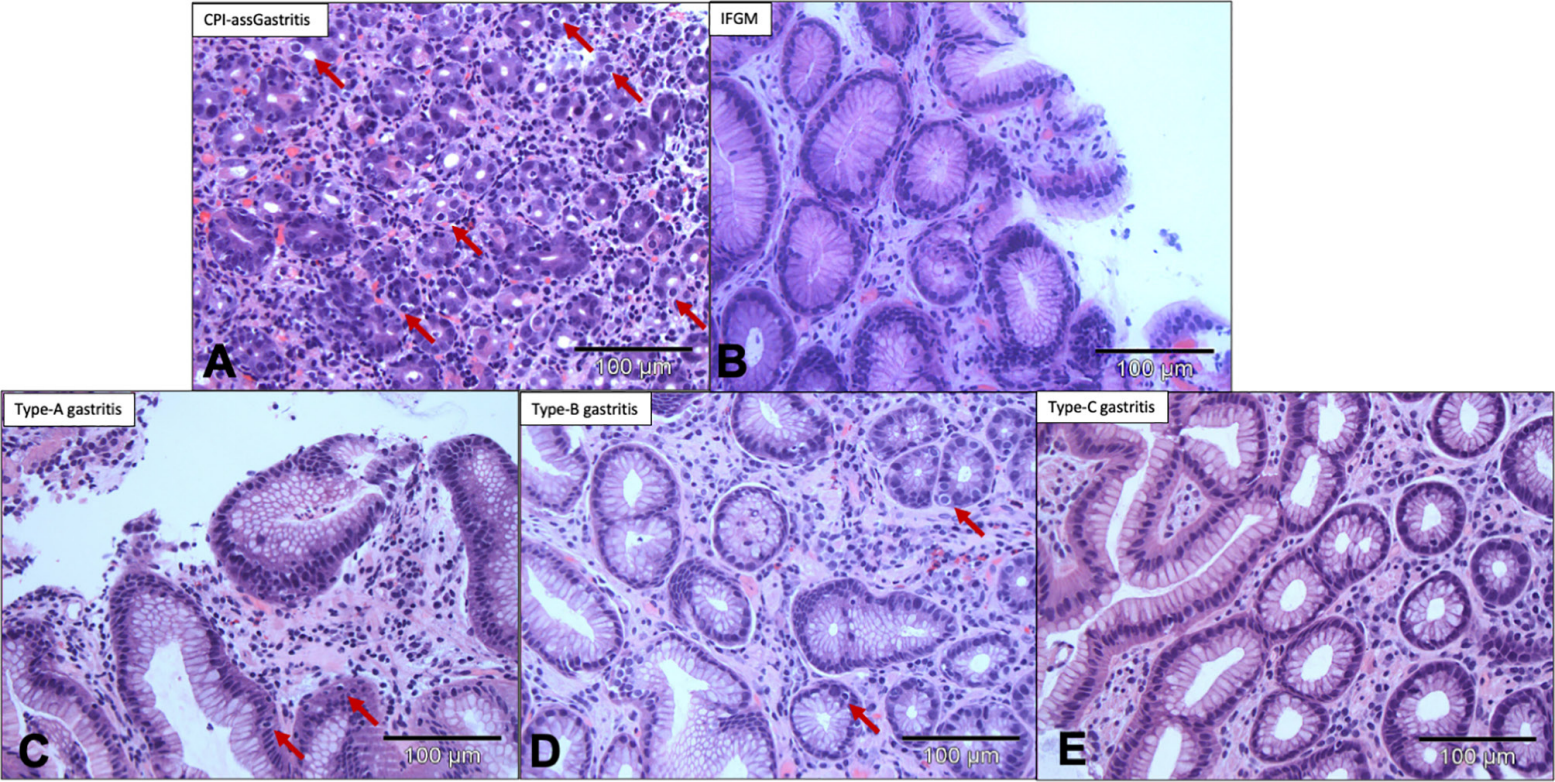
Conventional H&E staining suggests significant differences in frequency of apoptosis between CPI-associated gastritis and common types of gastritis. **(A)** Histological H&E image of CPI-associated gastritis shows numerous suspected apoptotic events in the glandular epithelia. **(B)** IFGM without suspected apoptotic events, **(C)** Type-A and **(D)** type-B gastritis with isolated suspected apoptotic events in the glandular epithelia. **(E)** Type-C gastritis with no suspected apoptosis in the glandular epithelia. H&E staining at 200× magnification. →, apoptosis; CPI, checkpoint inhibition; H&E, hematoxylin&eosin staining; IFGM, Inflammation free gastric mucosa.

Routine H&E diagnostic staining showed evidence of numerous apoptotic events in the glandular epithelia of all five patients treated with CPI. Furthermore, a partly lymphocytic, partly granulocytic inflammatory infiltrate with pronounced foveolar abscesses was found. *Helicobacter pylori* was not detected in any of the CPI patients’ biopsies in Giemsa staining but was found in five patients (all type-B gastritis) in the control group. In contrast, patients in the comparison cohort with IFGM showed no apoptotic features, patients with common A and B forms of gastritis only showed evidence of isolated apoptotic features in routine HE staining. No apoptosis was found in the patients with type C gastritis. The histologic features of the distinct forms of gastritis are summarized in **Table 3**. Overall, it was not possible to definitively classify glandular epithelial cells as apoptotic by means of H&E staining alone as lymphocytes and granulocytes migrating into the gland can exhibit a very similar cell morphology (**Figure 2**).

**Table 3:** Histological features of the different forms of gastritis.

	Type A gastritis	Type B gastritis	Type C gastritis	CPI-ass Gastritis
**Inflammatory infiltrate**	Lymphoplasmacytic-dominant inflammatory infiltrate	Mixed inflammatory infiltrate with a usually superficial granulocytic component close to the crypt lumina	Lymphoplasmacytic inflammatory infiltrate, sparse amount of intermingling neutrophilic granulocytes possible	Mixed inflammatory infiltrate with lymphoplasmacytic and granulocytic component, foveolar abscess formation possible
**apoptosis**	Mild increase of apoptotic cells in the gland epithelia (average of 1 apoptotic cell per 10HPF)	Mild increase of apoptotic cells in the gland epithelia (average of 1.5 apoptotic cells per 10 HPF)	No increase of apoptotic cells in the gland epithelia	Increase of apoptotic cells in the gland epithelia, detachment of apoptotic cells and shift to the glandular lumen (average of 6 apoptotic cells per 10 HPF)
**Additional characteristics**	Reduction/loss of parietal cells and hypoplasia of the glandular body in corpus mucosa	Detection of Helicobacter pylori *via* conventional H&E- and Giemsa stain, or immunohistochemistry	Fibrotic antral changes, foveolar hyperplasia, smooth muscle fibers radiating to surface parts, potentially intestinal type goblet cell metaplasia and occurence of pseudo-Paneth cells.	Anti-caspase 3 immunohistochemistry helpful in detection of apoptotic bodies/cells.

HPF, High Power Field.

### Immunohistochemical Anti-Caspase-3 Staining Confirmed That the Number of Apoptotic Events Was Higher in CPI-assGastritis Than in Common Types of Gastritis

**Figure 3:**
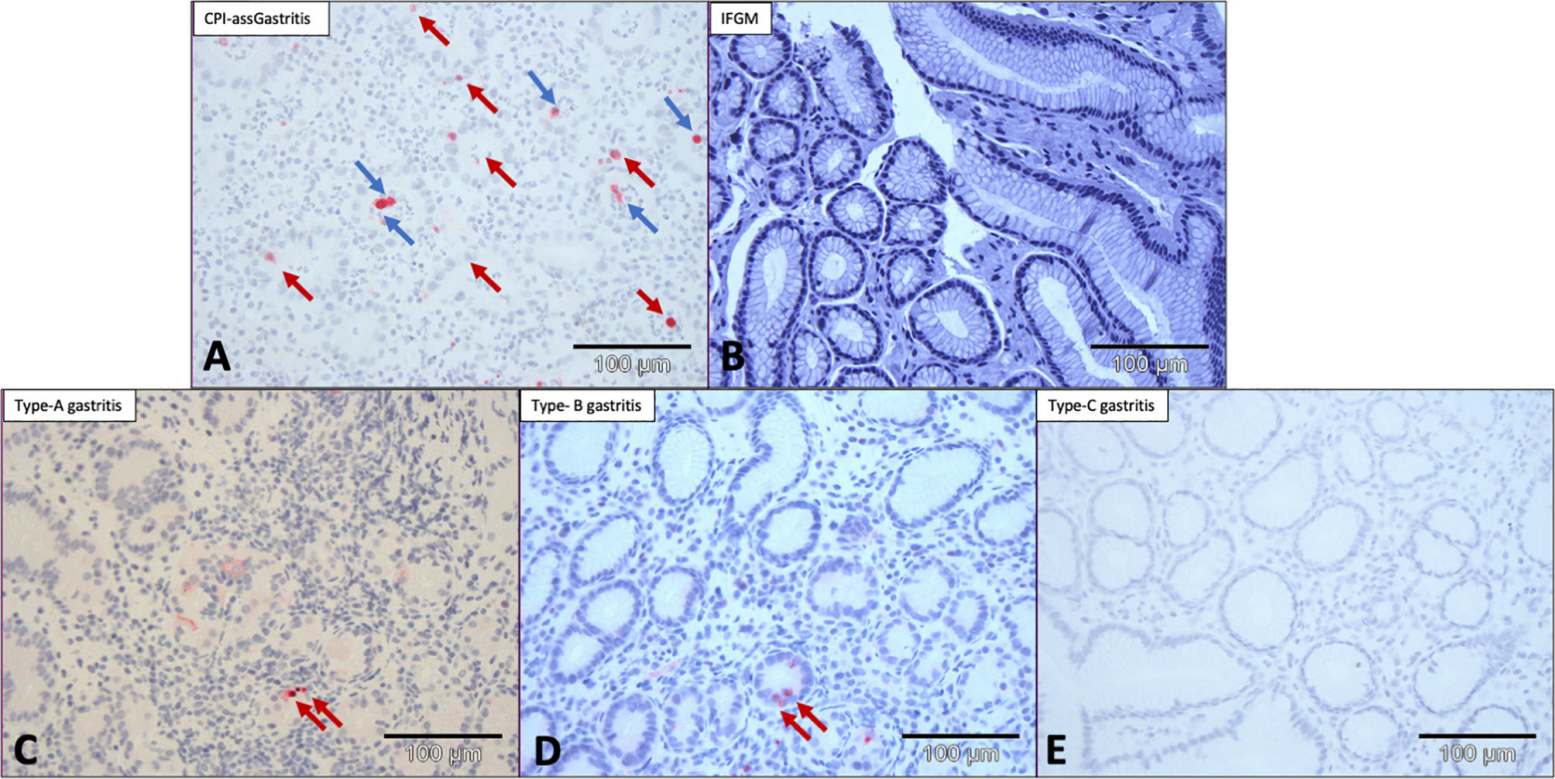
Anti-caspase-3 staining showing numerous apoptotic events in CPI-associated gastritis. **(A)** CPI-associated gastritis with numerous apoptotic events both in the glandular cell layer and glandular lumen. **(B)** IFGM without evidence of apoptosis. **(C)** Type-A and **(D)** type-B gastritis with only isolated apoptotic events in the glandular cell layer. **(E)** Type-C gastritis without evidence of apoptosis. Anti-caspase-3 staining at 200× magnification. → = apoptosis in glandular layer; →, apoptosis in glandular lumen; CPI, checkpoint inhibition; IFGM, Inflammation free gastric mucosa.

Immunohistochemical anti-caspase-3 staining was performed to identify apoptotic events. In patients with CPI-assGastritis, staining showed numerous apoptotic events both in the cell layer of the glandular epithelia and in detached cells shifted to the lumen of the gland (**Table 3**). In comparison, patients with IFGM showed no apoptosis, patients with type A and type B gastritis showed only isolated apoptotic features in the glandular cell layer and no detached apoptotic cells in the glandular lumen. No apoptosis was observed in patients with type C gastritis (**Figure 3**).

**Figure 4:**
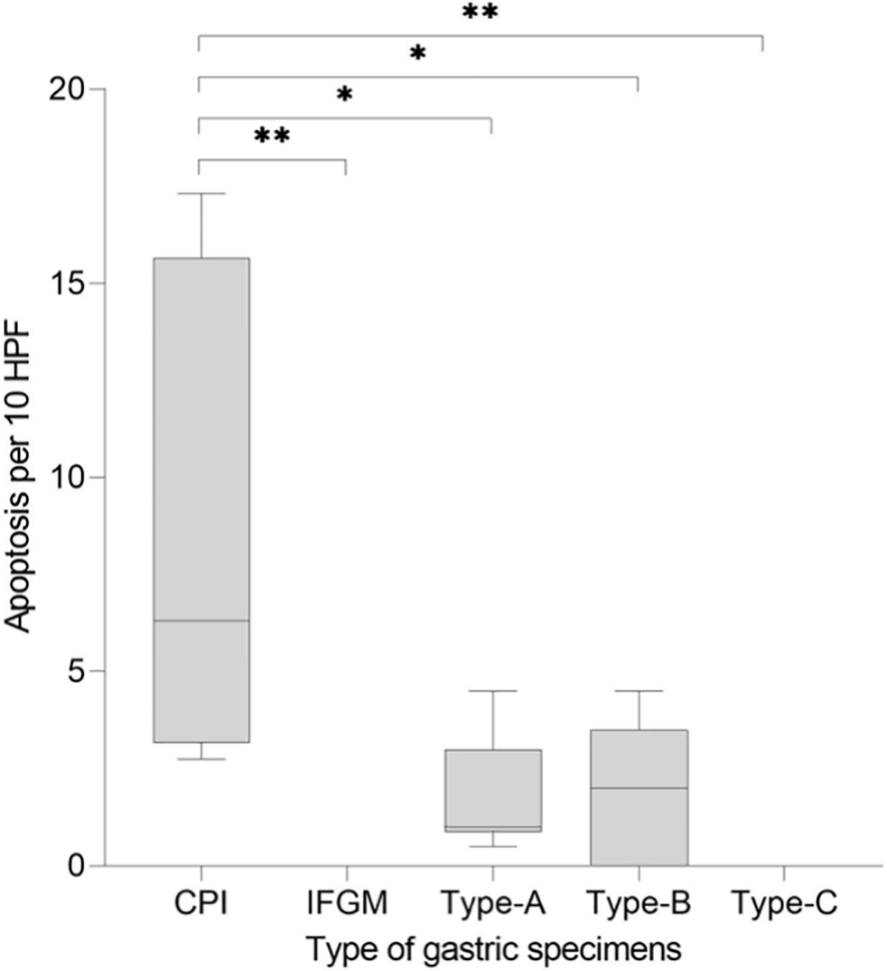
Comparison of CPI-associated gastritis with common forms of gastritis regarding apoptosis rate. The y-axis shows the number of apoptotic events per 10 HPF. The x-axis shows the different types of gastritis (CPI-associated, IFGM, type A, type B, and type C). CPI-associated gastritis shows a significantly higher apoptosis rate. CPI, checkpoint inhibition; HPF, high-power field; IFGM, Inflammation free gastric mucosa; *p < 0.05; **p < 0.01.

Manual counting of caspase-3-positive cells revealed a median of 6 apoptotic events/10 HPF (95% CI, 2.75–17.30) in patients with CPI-assGastritis, no apoptosis in patients with IFGM, a median of 1 apoptotic event/10 HPF (95% CI, 0.5–4.5) in patients with type A gastritis, a median of 2 apoptotic events/10 HPF (95% CI, 0–4.5) in patients with type B gastritis, and no apoptosis in patients with type-C gastritis. Patients with CPI-assGastritis had a significantly higher number of apoptotic events than patients with IFGM (p<0.01) type A gastritis (p<0.05), type-B gastritis (p<0.05), and type-C gastritis (p<0.01; **Figure 4**).

### Management of CPI-assGastritis and Further Clinical Course

All CPI-assGastritis patients received systemic treatment with high-dose corticosteroids. Four patients received 1 mg methylprednisolone per kg bodyweight: three patients intravenously (i.v.) and one per os (p.o.). One patient received 8 mg i.v. dexamethasone per day. Furthermore, all patients were treated with 40 mg pantoprazole twice per day. Metoclopramide and dimenhydrinate were administered intravenously to three of five patients as an antiemetic treatment. Treatment led to rapid improvement of clinical symptoms after a median of 6 days (range 2–7 days). Except for corticosteroids, no further immunosuppression was necessary. After resolution of symptoms, four of the five patients were subsequently rechallenged with PD1-based CPI. None of the patients had a second episode of CPI-assGastritis (**Table 2**).

### Selected Patient Cases Demonstrating the Diversity of the Clinical Picture of CPI-assGastritis

**Figure 5:**
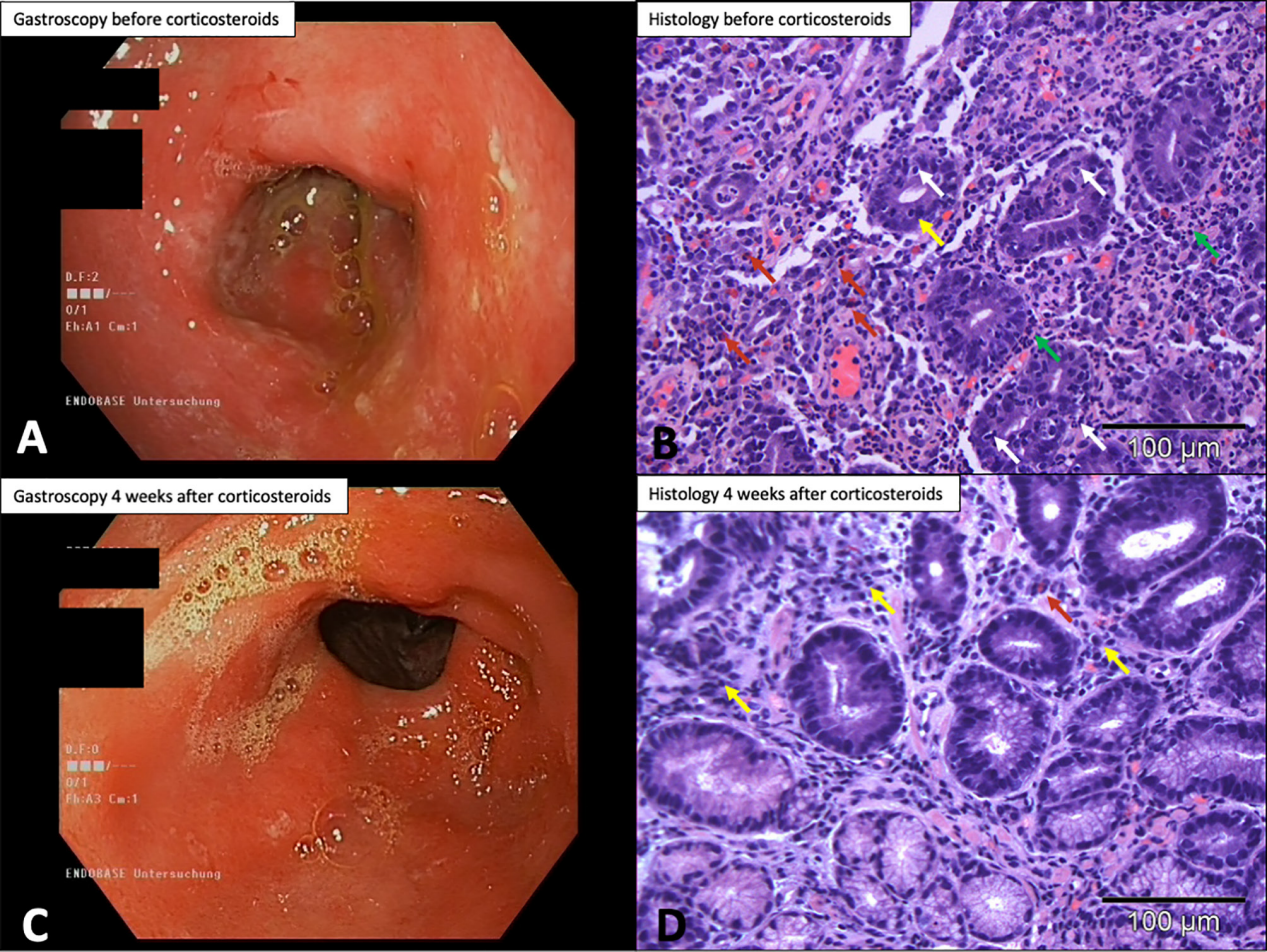
Case 1: gastroscopy and histologic findings. **(A)** Gastroscopy before initiation of corticosteroids with signs of erosive pangastritis. **(B)** Biopsy before corticosteroids with severe inflammation of the antral mucosa, lymphocyte infiltrates, eosinophilic and neutrophilic granulocytes, and numerous apoptotic events (H&E, 200× magnification). **(C)** Gastroscopy 4 weeks after corticosteroids showing marked improvement of inflammation and discrete prepyloric antrum gastritis. **(D)** Specimen biopsy 4 weeks after corticosteroids showing low lymphoplasmacytic cell infiltrate in the stroma and only isolated eosinophilic granulocytes (H&E, 200× magnification). H&E, hematoxylin&eosin staining; 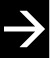, apoptosis; 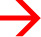, eosinophilic granulocytes; 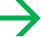, neutrophilic granuolocytes; 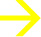, lymphocytes.

Case 1: A 38-year-old patient was diagnosed with stage IV melanoma with pulmonary metastases and iliac lymph node metastases. After receiving a total of 17 infusions of pembrolizumab in combination with epacadostat within a clinical trial (NCT02752074), the patient reported gastric pain, vomiting, and lack of appetite, as well as involuntary weight loss of 9 kg over 3 weeks. Gastroscopy showed signs of erosive pangastritis with distinct, diffusely erythematous as well as edematous mucosa throughout the stomach. The patient also had numerous confluent shallow erosions (maximum diameter: approximately 5 mm) throughout the stomach without evidence of previous bleeding (**Figure 5A**). Several samples were taken during gastroscopy. Histologic analysis revealed severe chronic and active ulcerative inflammation with infiltrates of lymphocytes, plasma cells, and neutrophils (**Figure 5B**). Treatment with 1 mg p.o. methylprednisolone per kg body weight and 40 mg pantoprazole i.v. twice daily was immediately started. This resulted in marked improvement of the patient’s clinical symptoms after 3 days. Four weeks later, a follow-up gastroscopy with histopathological analysis of biopsies was performed; this showed only a discrete gastritis in the prepyloric antrum with histologic features of a low-grade active gastritis (**Figures 5C, D**). After disease progression, the patient was treated with nivolumab in combination with relatlimab (NCT01968109) without recurrence of CPI-assGastritis.

**Figure 6:**
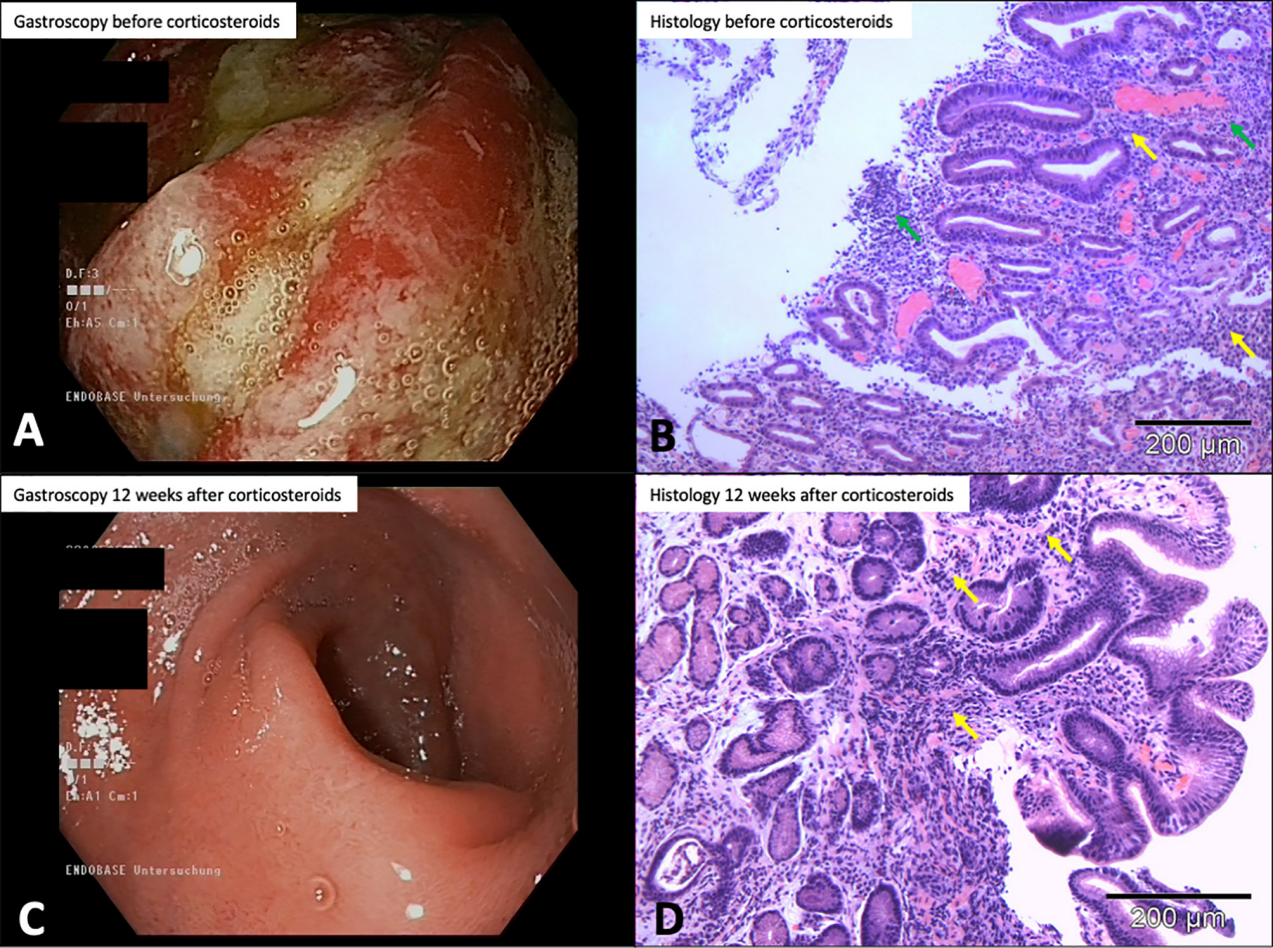
Case 2: gastroscopy and histologic findings. **(A)** Gastroscopy before corticosteroids showing marked erythematous pangastritis. **(B)** Sample biopsy before corticosteroids showing partly lymphocytic, partly granulocytic inflammatory infiltrate with superficial ulceration, hyperemia, and vascular ectasia (H&E, 100× magnification). **(C)** Gastroscopy 12 weeks after initiation of therapy showing marked improvement of chronic pangastritis. **(D)** Trial biopsy after therapy shows less stromal infiltrate than **(C)**, but a small patchy inflammatory infiltrate of the glandular epithelium remains (H&E, 100× magnification). H&E, hematoxylin&eosin staining, →, neutrophilic granuolocytes; →, lymphocytes.

Case 2: A 45-year-old male patient with stage IV melanoma who had received four doses of ipilimumab and nivolumab followed by two doses of nivolumab for bipulmonary metastases presented with severe heartburn, lack of appetite, nausea, and weight loss of approximately 10 kg over 3 weeks. Because the patient was no longer able to achieve adequate food intake, he was admitted to hospital for further evaluation. Subsequent gastroscopy revealed pronounced erythematous pangastritis with marked diffuse, patchy-to-streaky and edematous mucosa with prominent fibrinous deposits throughout the stomach (**Figure 6A**). Sample biopsies taken during the gastroscopy histologically showed ulcerative high-florid pangastritis (**Figure 6B**). CPI-assGastritis was suspected, and the patient therefore received i.v. methylprednisolone (1 mg/kg/day), i.v. pantoprazole (40 mg twice per day), and—because intake of solid food was no longer possible—a liquid diet. After 5 days there was a noticeable improvement, and the patient was able to eat solid food again. After 9 days in hospital, the patient could be discharged. Follow-up gastroscopy performed 3 months later revealed an overall improvement but ongoing presence of chronic pangastritis with marked diffuse, patchy reddened and edematous mucosa throughout the stomach (**Figure 6C**). Specimen biopsies histologically showed moderate to severe chronic gastritis with predominantly lymphoplasmacytic cells and occasional neutrophils (**Figure 6D**). Subsequent staging showed progressive disease, and the patient received pembrolizumab in combination with the histone deacetylase inhibitor domatinostat (NCT03278665) without further recurrence of CPI-assGastritis. Three months later the patient died from his progressive disease.

## Discussion

CPI-assGastritis is currently a rare but serious side effect of immune checkpoint blockade, and early diagnosis is often difficult because of nonspecific symptoms and the lack of clear histopathological classification. In our analysis, we examined the number of apoptotic events in the glandular epithelia of the stomach in patients with CPI-assGastritis and compared them for the first time with common type A, B, and C gastritis. We found a significantly higher rate of apoptosis in patients with CPI-assGastritis than in patients with common types of gastritis, which highlights the value of classic histopathology in combination with anti-Caspase 3 IHC.

Clinically, patients in our cohort with CPI-assGastritis showed a heterogeneous pattern of disease with a wide spectrum of epigastric symptoms ranging from mild malaise and nausea to severe epigastric pain and vomiting. In contrast to previously published case series, 60% of our patients experienced extreme weight loss (9, 11, 15, 17, 18). In the present study, all patients received dual CPI before the development of gastritis. This finding is highly consistent with observations in the literature. First, high-grade ir-AEs of grade 3 and 4 are known to occur significantly more frequently in melanoma patients on dual CPI of ipilimumab and nivolumab (58%) than in those receiving anti-PD1 monotherapy (23%) (19). Also, in the largest case collection on CPI-assGastritis to date, which studied a total of 12 patients, most patients received dual CPI (15). However, the development of CPI-assGastritis is not solely associated with dual CPI. Several case reports in the literature describe gastritis after anti-PD1 monotherapy (11, 17, 18). Nevertheless, it is important for oncologists to be aware that dual CPI certainly increases the likelihood of developing CPI-assGastritis, especially because new indications for dual CPI are currently advancing rapidly. In addition to melanoma, dual CPI is now approved for renal cell carcinoma and non-small cell lung carcinoma, and additional approvals may soon follow (4–6).. Therefore, severe cases of CPI-assGastritis are expected to become more common in oncology patients in the near future. Early detection of CPI-assGastritis is essential for the patient, because high-dose steroid therapy is indicated, whereas it is absolutely contraindicated for the other common forms of gastritis.

Because macroscopic appearance in gastroscopy does not enable reliable diagnosis of CPI-assGastritis, histopathological differentiation is of utmost importance. In particular, the typical histopathological pattern with a high rate of apoptoses observed in CPI-associated gastrointestinal side effects may help to distinguish CPI-assGastritis from other forms of gastritis. In contrast to previous studies on CPI-assGastritis in which apoptosis was quantified by means of H&E staining (15), we used immunohistochemical anti-caspase-3 staining, by which apoptosis can be reliably detected. The pattern of increased apoptosis rate has long been described for CPI-associated colitis (13, 14, 20). Another disease associated with increased apoptosis rates in the context of inflammation of the gastrointestinal tract is graft-versus-host disease (GvHD) (21–23). This raises the exciting question of whether the gastrointestinal side effect associated with CPI is similar to that seen in GvHD on the molecular level. The hypothesis of a molecular pathological relationship between the two diseases is supported not only by pronounced apoptosis rate, but also by the presence of a distinct inflammatory infiltrate consisting of extended lymphocytes, granulocytes, and cryptic or foveolar abscesses (9, 15, 20, 22, 24). Furthermore, CPI-associated gastrointestinal side effects and GvHD have similar treatment regimens consisting of immunosuppression in conjunction with primarily high-dose corticosteroids (25, 26).

Limitations of our study are its retrospective character and small cohort size. In addition, quantification of apoptosis was performed subjectively by hand counting rather than digital quantification. Unfortunately, apoptotic cells are physiologically present in the lumen of gastric glands during inflammatory processes, so they should not be used for quantification in CPI-assGastritis and hand counting is more reasonable. Nevertheless, due to the high clinical relevance of this topic and lack of information from randomized studies, we consider it important to report the clinical and histopathological observations of patients with CPI-assGastritis.

In conclusion, CPI-assGastritis is a challenge for the treating oncologist in terms of correct and early diagnosis. In patients treated with CPI, CPI-assGastritis should always be considered as the cause of unexplained epigastric symptoms. Diagnostic gastroscopy with specimen biopsies for histologic evaluation should be performed as soon as possible. The number of apoptotic events might help to histologically differentiate CPI-assGastritis from common type A, B, and C gastritis, and should be included in the pathology report when CPI-assGastritis is suspected. In this context, anti-caspase-3 immunohistochemistry is a helpful tool to precisely detect the extent of apoptotic cells. It is expected that this clinical picture, which is currently rarely observed, will become more common in the near future due to the increasing use of dual CPI for different tumor types.

## Data Availability Statement

The raw data supporting the conclusions of this article will be made available by the authors, without undue reservation.

## Ethics Statement 

The study was approved by the ethics committee of Duisburg-Essen University and conducted in accordance with the Declaration of Helsinki. Human biological samples and related data were provided by the Westdeutsche Biobank Essen (WBE, Essen University Hospital, University of Duisburg-Essen, Essen, Germany, approval number 19-8705-BO). The patients/participants provided their written informed consent to participate in this study.

## Author Contributions

J-MP and LZ analyzed and interpreted the patient data and were major contributors to writing the manuscript. JR and HR performed the histological examinations. JR-A was responsible for the gastroscopy. All authors contributed to the article and approved the submitted version.

## Conflict of Interest

J-MP served as consultant and/or has received honoraria from Bristol-Myers Squibb and Novartis, and has received travel support from Bristol-Myers Squibb, Novartis and Therakos. HR is on the advisory board of Bristol-Myers Squibb, received honoraria from Roche and Bristol-Myers Squibb, received travel support from Philips, Roche, and Bristol-Myers Squibb, received grants from Bristol-Myers Squibb and holds shares of Bayer. EL served as consultant or/and has received honoraria from Amgen, Actelion, Roche, Bristol-Myers Squibb, Merck Sharp & Dohme, Novartis, Janssen, Medac, Sanofi, and Sunpharma, and travel support from Amgen, Merck Sharp & Dohme, Bristol-Myers Squibb, Amgen, Pierre-Fabre, Sunpharma, and Novartis, outside the submitted work. SU received grants, personal fees, and non-financial support from Novartis, grants and non-financial support from Bristol-Myers Squibb; personal fees and non-financial support from Roche, personal fees from Merck Sharp & Dohme, and non-financial support from Amgen, outside the submitted work. DS received grants and other support from Bristol-Myers Squibb, personal fees from Bristol-Myers Squibb during the conduct of the study, personal fees from Amgen, personal fees from Boehringer Ingelheim, personal fees from InFlarX, personal fees and other support from Roche, grants, personal fees and other support from Novartis, personal fees from Incyte, personal fees and other support from Regeneron, personal fees from 4SC, personal fees from Sanofi, personal fees from Neracare, personal fees from Pierre-Fabre, personal fees and other support from Merck-EMD, personal fees from Pfizer, personal fees and other support from Philogen, personal fees from Array, personal fees and other support from MSD Sharp & Dohme, outside the submitted work. AR reported grants from Novartis, Bristol Myers Squibb, and Adtec, personal fees from Merck Sharp & Dohme, and nonfinancial support from Amgen, Roche, Merck Sharp & Dohme, Novartis, Bristol Myers Squibb, and Teva. LZ served as consultant and/or has received honoraria from Roche, Bristol-Myers Squibb, Merck Sharp & Dohme, Novartis, Pierre-Fabre, and Sanofi, research funding to institution from Novartis, travel support from Merck Sharp & Dohme, Bristol-Myers Squibb, Amgen, Pierre-Fabre, and Novartis, outside the submitted work.

The remaining authors declare that the research was conducted in the absence of any commercial or financial relationships that could be construed as a potential conflict of interest.

## Publisher’s Note

All claims expressed in this article are solely those of the authors and do not necessarily represent those of their affiliated organizations, or those of the publisher, the editors and the reviewers. Any product that may be evaluated in this article, or claim that may be made by its manufacturer, is not guaranteed or endorsed by the publisher.
